# Medical students’ research productivity and career preferences; a 2-year prospective follow-up study

**DOI:** 10.1186/s12909-017-0890-7

**Published:** 2017-03-03

**Authors:** Riitta Möller, Maria Shoshan

**Affiliations:** 1 Department of Medical Epidemiology and Biostatistics, Karolinska Institutet, Nobels väg 12 a, 171 77 Stockholm, Sweden; 2 Departments of Oncology-Pathology and Medical Epidemiology and Biostatistics, Karolinska Institutet, Stockholm, Sweden

**Keywords:** Medical students, Scholarly research, Undergraduate research, Student thesis, Research activities

## Abstract

**Background:**

Linking undergraduate medical education to scientific research is necessary for the quality of future health care, and students´ individual research projects are one way to do so. Assessment of the impact of such projects is of interest for both educational and research-oriented segments of medical schools. Here, we examined the scholarly products and medical students’ career preferences 2 years after a mandatory research project course.

**Methods:**

A prospective cross-sectional questionnaire study. All 581 students registered on a 20-week research project course between September 2010 through September 2012 were e-mailed a questionnaire 2 years after completing the course.

**Results:**

In total, 392 students (mean age 27 years; 60% females) responded (67% response rate). 59 students (15%) were co-authors on a scientific paper published in an international journal, 6 students had published in a national journal, and 57 students had co-authored a paper submitted for publication. Totally, 122 scientific papers had been submitted. Moreover, 67 (17%) students had given 107 oral or poster presentations nationally or internationally during the follow-up. Career-wise, 36 students (9%) had been registered as PhD students and an additional 127 students (34%) were planning to register. Those who did not plan doctoral studies were significantly older (*p* = 0.013) than those who did. However, 35% reported that they would in the coming 5 years prefer to work as clinicians only, and this group was significantly younger than those who envisaged participation in research. There were no significant gender differences.

**Conclusions:**

Approximately a third of the students had authored papers and/or public presentations, and a similar fraction had career plans involving a PhD degree. The results indicate that the project course had a positive impact on continued supervisor-student collaboration on a professional level, but also that strategies to encourage young doctors to perform clinical research may be needed.

## Background

The main purpose of undergraduate medical education is to train doctors in providing safe and effective patient-centered care. However, the rapid developments in healthcare and increase in the amount of information, as well as the easy access to it, demand that physicians make decisions based on reliable scientific evidence [1]. Therefore, linking research competencies to undergraduate education is necessary for the quality of future health care. Students’ individual research projects are one way to provide such training.

Research competencies may be broadly divided into 3 groups: generic competencies (e.g. the ability to synthesize findings and draw conclusions) and competencies related to “using research” (e.g. carry out a literature search and critically appraise evidence) or “doing research” (e.g. formulate a research question, collect and analyze own research data) [2, 3]. To develop these competencies, some universities offer students scholarly projects, which may be as short as a few weeks, either within the main curriculum or through extra-curricular activities [4–6]. They may comprise a short scientific project, or in-depth study in areas such as medical education, ethics and medical humanities, or health policy research [4, 5]. However, an increasing number of medical schools worldwide have individual projects performed in authentic research environments as a component of their curriculum [7, 8].

In addition to differences in training research competencies, the programs also differ with regard to the assessment of research skills. Shorter scholarly projects may be assessed as oral or poster presentations while students completing a substantial research project are often also assessed via a research report (sometimes called student thesis) [8, 9]. Consequently, academic outcomes, e.g., presentations and publications, have been measures of success of research project courses in undergraduate education. However, students´ projects are usually of limited scope and are in addition reported as individual work whereas medical science research papers need to fulfill a number of criteria regarding scope and significance, and are the result of teamwork. Thus, students´ reports as such rarely impact the scientific community. Nevertheless, their projects and data can be can be expected to be of interest for the overall science and the supervisors.

Students´ career choices are affected by, among other things, the differences in medical school entrance requirements, curriculum length and structure and postgraduate education structure [10, 11]. Some studies on the career choices of medical students required to perform a research project suggest that participation in research activities [12] and opportunity to publish research during training increases the likelihood of pursuing medical research [13–15]. However, none of the studies have had a prospective design, nor have they examined effects in specific research areas, or outcomes in terms of enrolment to PhD studies, and only a few have investigated gender issues [14].

This study aimed to examine the scientific outcomes of a mandatory undergraduate medical research project, as reflected by publications and scientific presentations, and to describe students’ career plans after the project work. Importantly, unlike several studies on optional research courses, we have studied a population that was unselected in terms of previous research interest and experience.

## Methods

### The context of the study

The context of this study is an undergraduate medical program, which consists of 11 bi-annual semesters, each comprising 20 weeks, and altogether corresponding to 5.5 academic years. The first 2 years cover mainly preclinical education (e.g. cell biology, anatomy, physiology) and the last 3.5 years mainly clinical education (e.g. medicine, surgery, pediatrics) including a total of 25 weeks of electives. Three so-called threads (professionalism, primary care and scientific education) run throughout the program. The 7^th^ semester is dedicated to a mandatory scientific research project (20 weeks) that can be done in a clinical or preclinical environment and results in a scientific report, written independently by the student. The overall aim is to provide students with deeper understanding of the scientific basis of medicine, and ability to interpret and evaluate scientific literature in order to become scientifically proficient clinicians. The authors of this paper (RM, MS) were directors and coordinator (MS) for the research project s during the time period under study. Supervisors are active researchers with at least a PhD degree. They receive 2300 euros as a bench fee to cover the expenses of student supervision. No financial support is given to the students or supervisors to cover conference attendance or the publication expenses. Optional co-supervisor(s) may be PhD students or other researchers in the same area.

As a final examination each student has to present an individual research report of about 20–35 pages in accordance with the university’s guidelines. Thus, the students are not allowed to present a manuscript intended to be submitted to a scientific journal. Students may, however, after the completed course continue the collaboration with their supervisors and publish their results as a scientific publication. They may also do extended research in parallel with undergraduate medical studies resulting in a dual clinical/higher research degree (MD/PhD). The scholarly results of such activities are not included in the current analysis.

### Study design and participants

This is a prospective cross-sectional questionnaire study. All 581 students registered on the research project course between September 2010 through September 2012 were eligible to participate. The students received oral and written information about the aim of the study stating that participation was voluntary and that declining to participate would not affect their education. The questionnaire was distributed by email 2 years after finished research project course, i.e. when the students had on average 2 semesters (1 year) left of their undergraduate medical studies.

The e-mail contained again written information of the aim of the study as well as a statement that by submitting the filled-in questionnaire the students gave their consent to participation in the study.

### Questionnaire

The questionnaire focused on scholarly activities after the finished course. Most of the questions were close-ended with a set of dichotomous answers. Interest in research and probability to do research in the future was ranked on a 5-point Likert scale, from 1 (considerably diminished; very unlikely) to 5 (considerably increased; very likely). An open-ended question concerning the reactions to the questionnaire was presented at the end of the questionnaire. Completion of the questionnaire took approximately 20 min. Each participant received 2 cinema tickets as compensation.

### Data analysis

Descriptive statistics were used to characterize and describe the sample features. For comparisons of differences between the groups Mann-Whitney or Kruskall-Wallis test was used where appropriate. The level of significance was set to 0.05. Bonferroni correction was used for multiple analyses. The statistical analyses were conducted with R version 3.1.

## Results

In total, 392 students (mean age 27 years; 60% females) returned the questionnaire corresponding to a response rate of 67%. The distribution of responses from the four semesters varied marginally. The majority of the students carried out a project in a clinical environment (*n* = 235; 60%) while the rest of the projects were classified as pre-clinical (*n* = 105; 27%), or as other, e.g. register studies, healthcare leadership, or medical education projects (*n* = 52; 13%).

### Co-authorship on a publication

Two years after they had completed the research project course, totally 61 (15.5%) students had published a scientific paper, 55 had published in an international paper, 2 students had published a paper in a national journal and 4 students had done both (Table 1). An additional 57 students were co-authors on a paper that had been submitted for publication. Thus, totally 122 scientific papers had been submitted within 2 years after the course and before completion of the entire medical education. Moreover, 21 students were co-authors of other professional publications such as clinical guidelines at their clinical placement. The publication frequency did not differ between research areas (clinical/pre-clinical/other) or site of the study (at or outside home university). Neither did gender or age group correlate with publication frequency (*p* > 0.05).

**Table 1:** A summary of submitted papers, publications, scientific presentations and conference attendance at the 2-year follow-up

	Total number of students (N)
Manuscripts submitted	122^a^
- Published in an international paper	55
- Published in a national paper	2
- Published in both international and national paper	4
- A paper submitted for publication	57
Authors on clinical guidelines	21
Scientific presentations	67
- At international meetings	25
- At national meetings	20
- At both international and national meetings	22
- At workplace	77
Attended a scientific conference without presentation	
-An international scientific conference	46
- A national scientific conference without presentation	64

^a^Total number of submitted manuscripts

### Scientific presentations

In total 67 (17%) students had given 107 scientific presentations (oral or posters) nationally or internationally during the follow-up. 20 students gave at least one presentation at a national meeting, 25 students gave at least one presentation at an international meeting and 22 students had given both (Table 1). The number of scientific presentations did not differ between research areas (clinical/pre-clinical/other), nor sites of the study (at or outside home university). Neither did student gender or age group correlate with number of presentations (*p* >0.05).

Furthermore, 77 students (20%) had given a scientific presentation at the workplace where they carried out their research project. The male students had given significantly more workplace presentations than had the female students (*p* = 0.002) Likewise, students who had done pre-clinical projects (the majority were males) had given more workplace presentations than other students (*p* = 0.002).

Of all 61 students who were co-authors of a published paper, 31 (51%) had not given any scientific presentation either nationally or internationally. Of the remaining 30 (49%) students, 4 had given a presentation at a national meeting, 10 had given a presentation at an international meeting and 16 had given both. Of the 331 students without a publication, 37 (11%) students had given a presentation.

### Attended a scientific conference

Although attending a scientific conference pertaining to the student´s project but without presenting data is not strictly a scientific outcome of the project, it may nevertheless reflect a budding interest in a research career. In total, 46 students (12%) had attended an international conference and 64 students (16%) a national scientific meeting. There was no significant difference in conference attendance between males and females, and the frequency was about the same for younger (<27 years) and older students (27–40 years) (*p* > 0.05).

### Changes in the interest in research

Our data showed that 51% of the students reported that their interest in research increased during the project work while 31% reported decreased interest, and 18% reported unchanged interest (Table 2). During the period from the end of the course to follow-up, the interest in research increased among 49%, decreased among 14% and remained unchanged among 37% of the students.

**Table 2:** Changes in research interest during and after the individual research projects

		Changes in research interest during the course					
		*N* = 46	*N* = 74	*N* = 71^a^	*N* = 133^a^	*N* = 63^b^	
		*Decreased a lot*	*Some decrease*	*No change*	*Some increase*	*Increased a lot*	
Changes in research interest at follow- up	*Increased a lot*	2	1	4	15	25	# of students w increased interest (*N* = 191)
	*Some increase*	11	29	23	63	18	
	*No change*	14	33	38	45	14	No change (*N* = 141)
	*Some decrease*	8	9	6	9	6	# of students w decreased interest (*N* = 52)
	*Decreased a lot*	11	2	0	1	0	
		# of students w decreased interest in research (*N* = 120)		No change (*N* = 71)	# of students w increased interest in research (*N* = 196)		

*W* with

^a^one missing value

^b^two missing values

### Intentions to pursue research

Registering as a PhD student was interpreted as a manifest intention to pursue research. During the 2-year follow-up, in total 36 students (9%) had registered as PhD students and an additional 127 students (34%) were planning to register. Rates of PhD student registration, or plans to register, were similar between the research project areas (clinical/pre-clinical/other), the site of the study (at or outside home university), and the genders (*p* >0.05). However, those who during follow-up registered as PhD students or were planning to register were younger than those who were not (*p* = 0.03 and *p* = 0.013, respectively).

One third of the students (33%) who had not become PhD students reported it was unlikely or very unlikely that they would do research in the future. Those who answered that they are “very unlikely” to do research in the future were significantly younger (*p* = 0.007) than those who answered that it was “rather unlikely”. The intention to pursue research did not differ significantly between research areas (clinical/pre-clinical/other) or genders.

Finally, the students were also asked what they would most like to do 5 years into the future. In total, 189 students (48%) wanted to work as a clinician with part-time (<30%) research, 60 students (15%) were interested in a greater proportion (>30%) of research time and 138 students (35%) reported they would prefer to work only as clinicians. The rest (2%) wanted other jobs. The students who had carried out pre-clinical research projects were more interested in doing part-time research (<30%) than were other students (*p* = 0.004). The students who had carried out a clinical research project were least interested in doing research in the future. Once again, there were no statistical significant differences between male and female students.

## Discussion

Future physicians need to train research skills as well as scientific attitude in order to not only participate in clinical and pre-clinical research, but also to become competent practitioners. Therefore, undergraduate medical education needs to involve research-related activities [8, 16]. Based on a questionnaire filled in by students 2 years after they had completed a mandatory research project, we have here investigated medical students’ scientific interest and productivity as reflected by project-related publications and scientific presentations as well as the students’ future career plans. The results based on 392 responses (67%) show that in total 122 scientific papers had been submitted within 2 years after the course during the 2-year follow-up, and 15% of the students were co-authors on a scientific paper published in an international journal. Furthermore, 17% of the students (half of them students without a publication) had given a presentation at an international and/or a national scientific meeting. The interest in an academic career increased during the research period.

Peer-reviewed journal publications are generally considered to be an indicator of research productivity, and are thus one major metric of the “return on investment” in supporting medical student research [14]. Earlier reports from three US medical schools show that 40–75% of the students had published at least one paper after their research period, and about half had given a presentation at an extramural meeting [17–19]. However, these figures do not represent a percentage of the entire student body, and include students in intercalated programs, students who had taken an elective research course or had research experiences of varied length. The AAMC 2013 graduation questionnaire showed that 42% of US medical students who had participated in a mandatory or elective research project were co-authors of a research paper [20]. Furthermore, according to a recent meta-analysis an average of 30% (95% CI 0.19–0.44) of research performed by medical students resulted in a peer-reviewed journal publication [14]. By comparison, our results show that only half as many students had published a paper; however, 30% of our students had submitted a paper for publication. We believe that this apparently low result is at least in part due to the fact that this particular course requires students to write an individual report without co-authors and thus not a standard manuscript. Instead, they have remained in sufficient post-project contact with their research group to participate as authors on a submitted manuscript.

Another circumstance, which in addition increases the power of our study to assess the research interest after the course, is that in Sweden, this 20-week research project involving international standard research methodologies is mandatory for obtaining the M.D. degree. Students who are particularly interested in a research career are instead encouraged to apply to the biomedicine program leading to a M.Sc. but not to a M.D. degree or clinical work. Thus, unlike many other studies, we have here not studied a selection of medical students who have actively applied to a research project course. Our study population can thus be taken to represent a wide range of pre-course research interest. This may in turn in part be reflected in the considerable variability in the research productivity of the students in question or the interest shown regarding future research activity. It should also be stressed that the research projects studied here does not require publishable results and reports. Nevertheless, our results indicate that scientific commitment was sufficiently high, or sufficiently kindled, during the project course to lead to more than 120 submitted manuscripts from less than 400 students.

Several other factors may also explain why publication frequencies differ between different medical schools and countries [14, 17–20]. The time devoted to curricular research experiences varies from some weeks to some months but is seldom more than one semester (intercalated programs excluded) [14, 17]. Thus, even the longest research periods are relatively short for carrying out a research project from planning through data collection, analysis, writing and publishing. The intended outcomes for students’ research period also vary; some have a focus on the research process and methodology itself while others encourage students and their supervisors to write a publishable paper [6]. As indicated above, in the present study, the project course studied here has a clear societal focus, i.e., to produce better physicians with a good grasp of the scientific basis of modern medicine. Furthermore, as our study group represents unselected medical students and their experiences from a comparatively long research period, we believe that our results show a generalizable impact of an authentic research environment and methodology.

At the time of responding to the questionnaire, the students had on average 2 semesters (1 year) left of their undergraduate medical studies. That fairly many (9%) had nevertheless already been registered as PhD students is perceived as encouraging, as it has been a long-standing worry that clinical research is not sufficiently boosted by M.D.s with a Ph.D. degree [21].

We did not find any substantial differences between male and female students’ interest and involvement in research, which is quite encouraging. These results are in contrast to those of Funston et al. [22] who showed that significantly fewer female students expressed interest in research participation in the future. By contrast, Amgad et al. [14] found no apparent gender difference in involvement in research during medical school, interest in research career, attitudes towards research, or research knowledge and skills. However, they found that male students were significantly more likely to publish (OR = 1.59; 126–2.01) during medical school than female students. These data are based on mandatory as well as voluntary research projects, and it is not clear whether the latter have a male preponderance, in turn suggesting a greater pre-existing research interest. Again, as our study group is unselected in terms of such interest and of gender, our results suggest more clearly that male and female students had comparable research interest/activity after the project work.

The finding that the younger students were more interested in a Ph.D. degree, but were also the least interested in doing research in the future was unanticipated. Previous studies have mentioned financial worries, lack of supervision and encouragement and inflexible curricula as possible obstacles to a research career [14] but the literature is sparse regarding the impact of age. It is possible that clinical projects require a higher degree of independence than other projects since clinical supervisors seldom work fulltime with research and are not available on a daily basis wherefore the common meetings and other activities may have to be scheduled rather than integrated in the daily work. We speculate that young students may be troubled by the level of independence required, and/or that older students are more knowledgeable about career choices and the impact that research may have on their career development. The results thus indicate a need for a strategy to attract more young doctors to clinical research. One aspect to consider is how the quality of project supervision affects student attitudes to research; we are currently examining this.

One strength of the current study is the prospective design while almost all published studies about students’ interest in research are retrospective [14]. In addition, the response rate was good and the gender distribution among respondents corresponded to that of our medical school in general. We also included students from several terms to minimize peer group influence on reported experiences. An additional strength is that the study group is unselected in terms of pre-course interest in research. One weakness that we used self-reported data from the students and we cannot claim that the interest shown, or the lack of it, has a direct causal relationship to the course, since students who decide to perform research may already have a keen interest in research. Altogether, this study provides evidence that medical students have a considerable and evolvable research interest that is promising for the future development of clinical medicine as well as medical research.

## Conclusions

During the follow-up time, approximately a third of the students had authored papers and/or public presentations, and a similar fraction had career plans involving a PhD degree. The results indicate that an important outcome of the course is that the scientific collaboration of supervisors and students often continues on a professional level long after the course. Future studies should address the role of the supervisor but also that strategies to encourage young doctors to perform clinical research may be needed.

## Abbreviations

AAMC: The association of american medical colleges
MD: Medical doctor
PhD: Doctor of philosophy
